# Establishment and evaluation of an overlap extension polymerase chain reaction technique for rapid and efficient detection of drug-resistance in *Mycobacterium tuberculosis*

**DOI:** 10.1186/s40249-022-00953-5

**Published:** 2022-03-24

**Authors:** Jungang Li, Jing Ouyang, Jing Yuan, Tongxin Li, Ming Luo, Jing Wang, Yaokai Chen

**Affiliations:** 1 Central Laboratory, Chongqing Public Health Medical Center, Chongqing, China; 2 Clinical Research Center, Chongqing Public Health Medical Center, Chongqing, China; 3 Division of Infectious Diseases, Chongqing Public Health Medical Center, Shapingba District, 109 Baoyu Road, Chongqing, 400036 China

**Keywords:** SOE-PCR, *Mycobacterium tuberculosis*, Drug-resistance, Sequencing

## Abstract

**Background:**

Rapid and accurate detection of drug resistance in *Mycobacterium tuberculosis* is critical for effective control of tuberculosis (TB). Herein, we established a novel, low cost strategy having high accuracy and speed for the detection of *M. tuberculosis* drug resistance, using gene splicing by overlap extension PCR (SOE PCR).

**Methods:**

The SOE PCR assay and Sanger sequencing are designed and constructed to detect mutations of r*poB, embB, katG*, and *inhA* promoter, which have been considered as the major contributors to rifampicin (RFP), isoniazid (INH), and ethambutol (EMB) resistance in *M. tuberculosis*. One hundred and eight *M. tuberculosis* isolates came from mycobacterial cultures of TB cases at Chongqing Public Health Medical Center in China from December 2018 to April 2019, of which 56 isolates were tested with the GeneXpert MTB/RIF assay. Performance evaluation of the SOE PCR technique was compared with traditional mycobacterial culture and drug susceptibility testing (DST) or GeneXpert MTB/RIF among these isolates. Kappa identity test was used to analyze the consistency of the different diagnostic methods.

**Results:**

We found that the mutations of S531L, S315T and M306V were most prevalent for RFP, INH and EMB resistance, respectively, in the 108 M*. tuberculosis* isolates. Compared with phenotypic DST, the sensitivity and specificity of the SOE PCR assay for resistance detection were 100.00% and 88.00% for RFP, 94.64% and 94.23% for INH, and 68.97% and 79.75% for EMB, respectively. Compared with the GeneXpert MTB/RIF, the SOE PCR method was completely consistent with results of the GeneXpert MTB/RIF, with a concordance of 100% for resistance to RFP.

**Conclusions:**

In present study, a novel SOE PCR diagnostic method was successfully developed for the accurate detection of *M. tuberculosis* drug resistance. Our results using this method have a high consistency with that of traditional phenotypic DST or GeneXpert MTB/RIF, and SOE PCR testing in clinical isolates can also be conducted rapidly and simultaneously for detection of drug resistance to RFP, EMB, and INH.

**Graphical Abstract:**

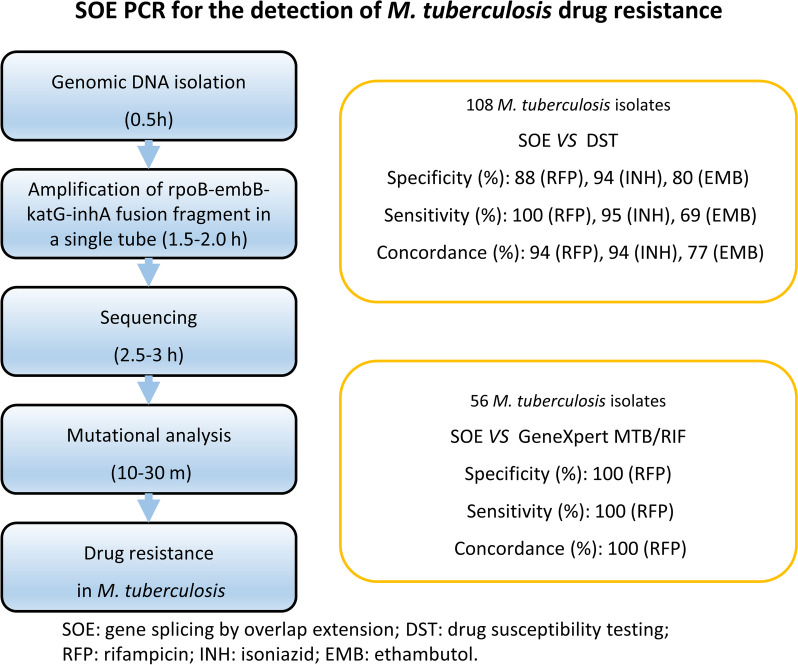

**Supplementary Information:**

The online version contains supplementary material available at 10.1186/s40249-022-00953-5.

## Background

Tuberculosis (TB) is an airborne infectious disease, caused by the bacillus, *Mycobacterium tuberculosis.* Until the emergence of the currently prevailing COVID-19 pandemic, as the leading infectious disease killer globally, an estimated 10 million cases developed TB, and 1.5 million people died of TB worldwide in 2020 [1]. Moreover, drug-resistant TB (DR-TB) presents an even more difficult challenge for control and management of tuberculosis (TB), especially in low-income countries. Globally in 2019, approximately half a million cases developed rifampicin-resistant TB (RR-TB), and among these cases, 78% were multidrug-resistant TB (MDR-TB) [1].

Rapid and accurate detection of DR-TB is the basis for relevant interventions of TB control programs, as well as allowing clinicians to tailor effective TB treatment strategies. China has the second largest share of the global burden of MDR-TB, and has a relatively low MDR-TB detection rate [1]. Increasing the detection rate of MDR-TB would obviously affect MDR-TB prevalence. It has been predicted, using the dynamic Markov model, that the prevalence of MDR-TB by 2050 would be greatly reduced (by an approximate 25.9 to 36.2% decline), if the MDR-TB detection rate in China increased from 22.5% to 50–70%, at around 5% per year [2]. In clinic, traditional mycobacterial culture and drug susceptibility testing (DST) remains the gold standard for the diagnosis of DR-TB [3]. However, DST may take up to several weeks to obtain a conclusive result for identification of *M. tuberculosis* drug resistance, which may unduly delay timeous critical decisions concerning patient treatment. In addition, the method based on culture requires trained personnel and high biosafety-level laboratories for the assurance of personal safety. Comparatively, genotypic drug-resistance detection techniques, including real-time fluorescent PCR testing [4], GeneXpert MTB/RIF [5], linear probe hybridization [6], DNA microarray technology [7] and gene chip (biochip) technology [8], have facilitated the rapid diagnosis of TB and also the detection of anti-tuberculous drug resistance to some extent; however, the relatively high cost, complexity of use, and the restricted accessibility associated with these techniques have limited their widespread application in general clinical practice [9–11]. The GeneXpert MTB/RIF test can rapidly identify *M. tuberculosis* bacilli and simultaneously detect drug resistance; nevertheless, it is limited to rifampicin resistance only [5]. Routine real-time fluorescent PCR testing can detect different drug-resistant-related gene targets, but it needs to be performed separately for each gene target. DNA microarray and gene chip technology allow genome-wide comparison of their genetic contents; however, the high cost required for the synthesis of gene-specific primers necessary to amplify each gene in a genome, and the dedicated software required to analyze the data, limit their application in routine clinical practice [7, 12].

Notably, gene splicing by overlap extension PCR (SOE PCR) is a simple, low-cost, and powerful strategy for cloning DNA sequences without the need for the numerous and labor-intensive digestion and ligation reactions, which string together individual segments of amplified DNA using homologous sequences added to the 5′-ends of specific primers [13]. The SOE PCR method can be utilized for simultaneous splicing of multiple DNA fragments in a single reaction tube, in which the reaction times and the quantities of raw materials that are otherwise required for conventional PCR processing, are significantly reduced [14, 15]. In the present study, in order to reinforce the arsenal used for diagnosing *M. tuberculosis*, and to further mitigate the limitations of currently available diagnostic technologies in a clinical setting, we established a novel strategy for the detection of *M. tuberculosis* drug resistance, using SOE PCR protocols and DNA sequencing. We assessed the clinical value of this PCR-based technique for detection of drug resistance to rifampicin (RFP), isoniazid (INH), and ethambutol (EMB), and compared this method with the DST method and the GeneXpert MTB/RIF assay in 108 clinical *M. tuberculosis* isolates.

## Methods

### Study design and setting

This was a diagnostic test assessment for detection of *M. tuberculosis* drug resistance conducted at a tertiary infectious diseases hospital, i.e., Chongqing Public Health Medical Center in China. The test method is the SOE PCR assay accompanied by Sanger sequencing, and the reference methods include phenotypic DST and GeneXpert MTB/RIF. One hundred and eight clinical *M. tuberculosis* isolates from December 2018 to April 2019 were selected to compare the SOE PCR assay with phenotypic DST for detecting RFP, INH, and EMB resistance. Among these specimen, 56 isolates which had GeneXpert MTB/RIF results were used to compare the SOE PCR assay for detecting RFP resistance.

### Selection criteria of isolates

*M. tuberculosis* isolates with different resistance properties were selected. The selection criteria were as follows: (a) Strains were isolated from sputum of TB patients and positively identified as *M. tuberculosis* according to standard laboratory procedures [16]; (b) Results of phenotypic DST for RFP, INH, and EMB resistance were complete and traceable, among which the specimens with GeneXpert MTB/RIF results would be compared with the SOE PCR assay; (c) The strains should have different resistance properties, including drug sensitive isolates and those resistant to one or more drugs.

### Reference methods

Phenotypic DST and GeneXpert MTB/RIF were used as the reference methods as they have been considered as the standard methods for the diagnosis of DR-TB [3]. Phenotypic DST was carried out according to standard guidelines using the L–J proportional method, for RFP, INH, and EMB [16]. GeneXpert MTB/RIF was performed according to the standard protocol [5].

### Reagents and instrumentation for the SOE PCR assay

The 10 × Buffer, dNTPs, and hot-start Taq polymerase used in the PCR reaction were purchased from the Nanjing Nazyme Biotech Company (Nanjing, China), Lowenstein–Jensen medium was purchased from the Zhuhai Yinke Company (Zhuhai, China), and the primers were synthesized by Beijing Tsingke Biotech Co., Ltd., Chongqing Branch (Chongqing, China).

The major laboratory instruments used in this study included the Eppendorf ThermoMixer C metal bath (Eppendorf AG, Germany), the ETC811 PCR instrument (Eastwin Life Sciences Inc; Beijing, China), the DYY-16D electrophoresis instrument (Beijing Liuyi Instruments; Beijing, China), the ChampGel gel imager (China Saizhi Company; Beijing, China), and the GeneXpert MTB/RIF assay (Cepheid; Sunnyvale, CA, USA).

### Primers for the SOE PCR assay

Using the DNA sequence of the laboratory reference strain of *M. tuberculosis* (H37Rv) as a template, we designed PCR primers, the amplifying products of which would contain the mutation sites of the *rpoB, embB, katG*, and *inhA* promoters. These genes confer resistance to RFP, INH, and EMB. The specific sequences are shown in Table 1.

**Table 1:** Primer sequences for the SOE PCR assay

Gene/region	Primer	Sequence (5′–3′)
*ropB*	*rpoB*_For	GTACGGTCGGCGAGCTGATCCA
	*rpoB*_Rev	CACCGGGTGCACGTCGCGGACCTCCAGCCCGGCACGCTCACGTGACAGAC
*embB*	*embB*_For	GGAGGTCCGCGACGTGCACCCGGTGATATTCGGCTTCCTGCTC
	*embB*_Rev	ACGGAAGGGATCCTCCGGGCTGCCGAACCAGCGGAAATAGTTGGA
*katG*	*katG*_For	CGGCAGCCCGGAGGATCCCTTCCGTATGGCACCGGAACCGGTAA
	*katG*_Rev	ACGCAAGCGCCAGCAGGGCTCTTCGTCAGCTCCCACTCGTAGCCGTACA
*inhA* promoter	*inhA*_For	GACGAAGAGCCCTGCTGGCGCTTGCGTAACCCCAGTGCGAAAGTTCCCG
	*inhA*_Rev	GGACTGAACGGGATACGAATGG
	TBseq (Sequencing primer)	ACCAGATCCGGGTCGGCATG

### The principle of the SOE PCR assay

Four major drug-resistance-related gene fragments, i.e., *rpoB*, *embB*, *katG*, and the *inhA* promoter, were ligated and amplified into a fusion fragment of approximately 700 base pairs (bp) in a single reaction tube, after which the four drug-resistance-related gene fragments were sequenced via Sanger sequencing in one read, greatly accelerating the detection of the presence of drug-resistance mutations that may exist in a specific *M. tuberculosis* isolate.

**Fig. 1:**
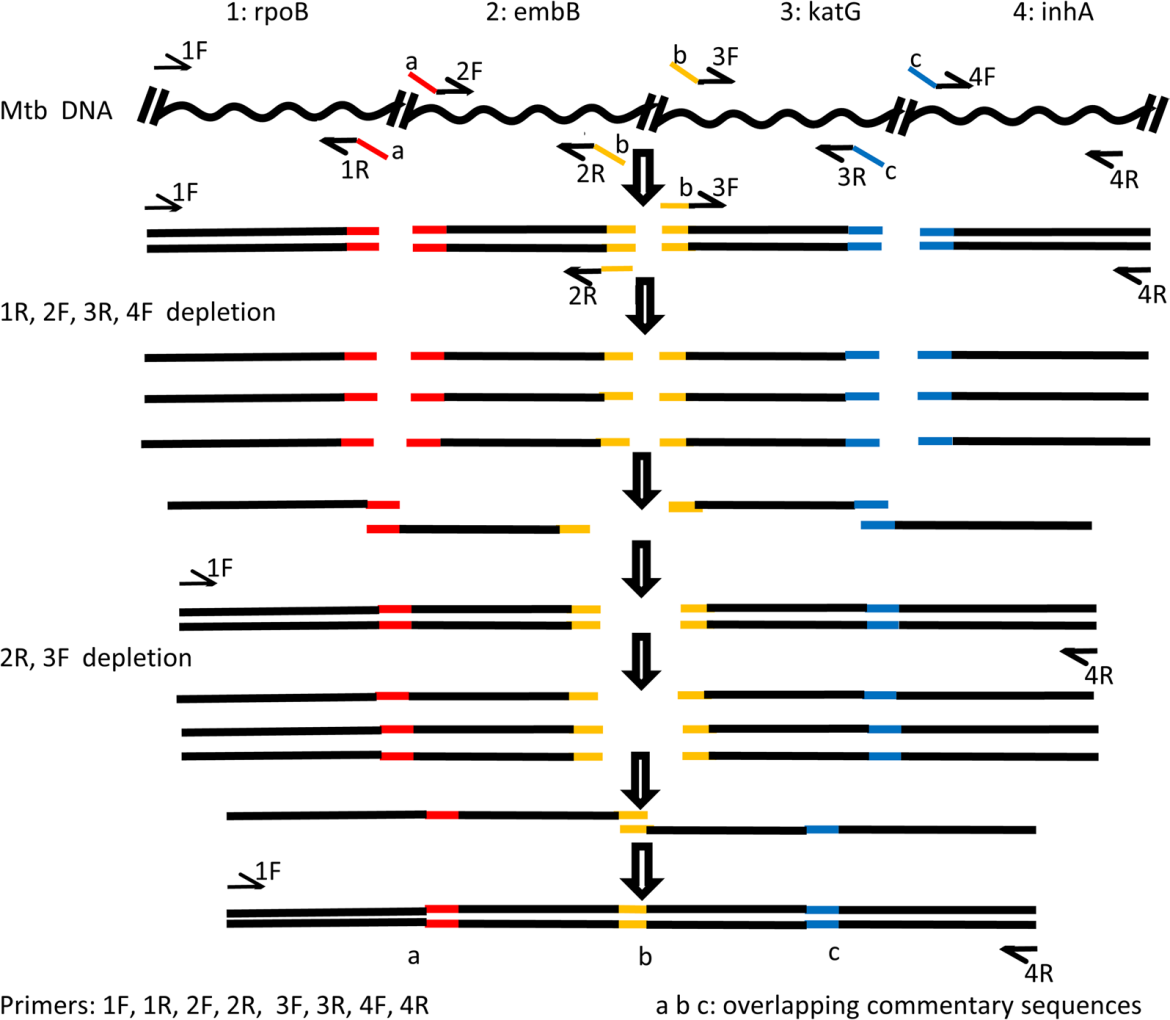
Schematic diagram of the principle of SOE PCR. SOE PCR: Gene splicing by overlap extension PCR

There were three different overlapping complementary sequences between the primers *rpoB*_Rev (1R) and *embB*_For (2F), between primers *embB*_Rev (2R) and *katG*_For (3F), and between primers *katG*_Rev (3R) and *inhA*_For (4F) respectively, as indicated in different colors in Fig. 1. In the SOE PCR reaction system, the concentrations of the primers 1F and 4R is higher than that of the other six primers, while the concentrations of the primers 2R and 3F is higher than that of the primers 1R, 2F, 3R, and 4F.

At the initial stage of the SOE PCR reaction, double-stranded DNA fragments of *rpoB, embB, katG,* and *inhA* were produced. As the reaction cycles increase, the primers 1R, 2F, 3R, 4F are depleted and exhausted, and the single-stranded DNA fragments of *rpoB, embB, katG,* and *inhA* are produced. During this process, there is a probability of gene splicing between the fragments *rpoB* and *embB*, and between the fragments *katG* and *inhA*.

Then, as the reaction cycles increase, the primers 2R and 3F are also depleted and exhausted, and the single-stranded DNA fragments of spliced *rpoB-embB* and spliced *katG-inhA* are produced. During this process, there is a probability of gene splicing between the spliced rpoB-embB and the spliced katG-inhA.

Once a small amount of the *rpoB-embB-katG-inhA* fusion fragment is produced in the SOE PCR system, it can then be used as a template to be amplified by primers 1F and 4R at both ends. The principle of SOE PCR in this study is illustrated in Fig. 1.

### The detection of drug resistance in clinical M. tuberculosis isolates using the SOE PCR assay

H37Rv, the laboratory reference strain of *M. tuberculosis,* which is sensitive to all anti-tuberculous drugs and has no gene mutations identifiable as drug-resistance genes, was used as the wild-type control. Crude genomic DNA was isolated from freshly cultured bacteria via the rapid boiling method. Briefly, the bacterial cells were suspended with Tris–EDTA buffer (pH 8.0). Then, 100 μl suspensions at a concentration of 10^8^ cells/ml were heated at 100 °C for 30 min. The cellular debris was removed by centrifugation at 12,000 g for 5 min. The genomic DNA in the supernatant was then used as the PCR template.

The extracted DNA from 108 clinical *M. tuberculosis* isolates was used for the amplification of the *rpoB-embB-katG-inhA* fusion fragment via the SOE PCR technique, and physiological saline was used as a negative control. Since naturally-occurring *rpoB-embB-katG-inhA* fusion fragments do not exist in nature, it was not applicable to set up a positive control in this instance.The 50 μl PCR reaction system contained 5.0 μl of 10 × Buffer, 5.0 μl of 10 μmol/l primers, 5.0 μl of template DNA, 1.0 μl of 2.5 mol/l dNTPs, 0.2 μl of 5 U/μl *Taq* polymerase, and 33.8 μl of ddH_2_O. The PCR procedure was set as follows: Step 1: 95 °C for 5 min; Step 2: 95 °C for 10 s; Step 3: 68 °C, reduce 0.5 °C for each cycle for 10 s; Step 4: 72 °C for 30 s; Step 5: 95 °C for 10 s, Step 6: 59 °C for 10 s, Step 7: 72 °C for 30 s; Steps 2–4: 30 cycles, and Steps 5–7: 40 cycles. Thus, the total duration for the SOE PCR amplification process would be around 2 h.

The *rpoB-embB-katG-inhA* fusion fragment obtained by SOE PCR amplification was sequenced using primer TBseq. This step of the process is likely to consume less than 5 h. The sequencing results of the *rpoB-embB-katG-inhA* fusion fragments from the 108 clinical isolates, together with the DNA sequence of the standard strain (H37Rv), were then imported into MegAlign for comparative analysis to search for the associated drug resistance mutations. The DNA sequence of the *rpoB* (1–273 bp)-*embB* (274–420 bp)-*katG* (421–564 bp)-*inhA* (565–722 bp) fusion fragment obtained by the SOE PCR technique is shown in Additional file 1. The amino acid sequence associated with the *rpoB-embB-katG-inhA* fusion DNA fragment sequence of the standard strain H37Rv is shown in Additional file 2 (*inhA* promoter encodes no amino acid).

The principle of mutational analysis is as follows:

1. The major mutation sites in the rpoB amino acid sequence included rpoB 511, 513, 515, 516, 518, 519, 526, 531, and 533, corresponding to amino acid mutation sites 51, 53, 55, 56, 58, 59, 66, 71, and 73 in the rpoB-embB-katG-inhA amino acid sequence. Table 2 illustrates the corresponding relationship of the major mutation sites between the rpoB amino acid sequence and the amino acid sequence of the fusion fragment.
2. Amino acid residue site 306 in embB corresponded to residue site 116 of the fusion fragment, mutation of which may cause EMB resistance.
3. Amino acid residue site 315 in katG corresponded to the amino acid residue site 153 of the fusion fragment, mutation of which may cause INH resistance.
4. Site-15 and site-8 of the inhA promoter DNA sequence corresponded to site 653 and site 660 of the DNA sequence of the *rpoB-embB-katG-inhA* fusion fragment, and mutations at these two sites cause INH resistance.

**Table 2:** Corresponding relationship of the major mutation sites between the rpoB amino acid sequence and the amino acid sequence of the fusion fragment

Amino acid residue sites of rpoB	Amino acid residue sites of the fusion fragment	DNA sites of fusion fragment
511	51	151–153
513	53	157–159
515	55	163–165
516	56	166–168
518	58	172–174
519	59	175–177
526	66	196–198
531	71	211–213
533	73	217–219

### Data analysis

An appropriately-trained investigator used a 4-grid table to conduct Kappa identity test and to calculate the sensitivity, specificity, positive predictive value, negative predictive value, concordance, and Kappa value, comparing results of the SOE PCR method with phenotypic DST or GeneXpert MTB/RIF testing results. Kappa values below 0.2, 0.21–0.40, 0.41–0.60, 0.61–0.80, and above 0.8 are considered as slight, fair, moderate, substantial, and almost perfect agreement, respectively [17]. The data analysis was double-checked by another trained investigator and a statistician from the School of Biomedical Engineering, Capital Medical University, Beijing, China.

## Results

### Acquisition of different spliced gene fusion fragments

**Fig. 2:**
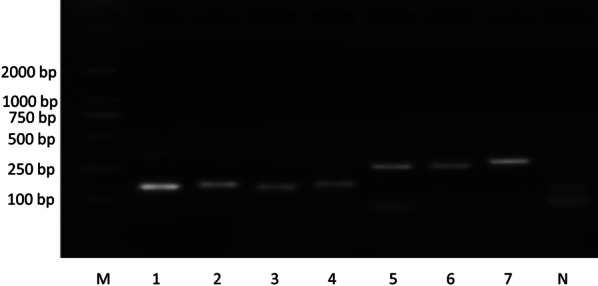
Results of agarose gel electrophoresis. M represents the marker, and N represents the negative control. 1–4 represent amplification products of the fragments of *rpoB, embB, katG*, and *inhA*, 5 represents the amplification product of the *rpoB-embB* fragment, 6 represents the amplification product of the *embB-katG* fragment, and 7 represents the amplification product of the *katG-inhA* fragment

As shown in Fig. 2, we successfully spliced *rpoB-embB*, *embB-katG* and *katG-inhA* fragments after optimization of the SOE PCR conditions, which indicates that the primer design was feasible.

### Performance evaluation of the SOE PCR technique in the detection of drug resistance in clinical *M. tuberculosis* isolates

**Fig. 3:**
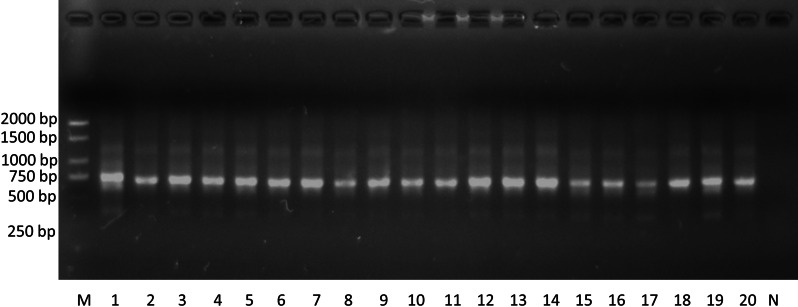
Gel electrophoresis results of SOE PCR products from DNA specimens of some clinical *M. tuberculosis* isolates. M is the DNA marker, 1–20 represent the amplified products, and N is the negative control

As shown in Fig. 3, the *rpoB-embB-katG-inhA* fusion fragments were successfully isolated from the amplified products via agarose gel electrophoresis. The results of comparative analysis showed that 64 clinical isolates had RFP resistance, 56 isolates had INH resistance, 36 isolates had EMB resistance. The mutations of S531L, S315T and M306V were most prevalent for RFP, INH and EMB resistance, respectively, in these M*. tuberculosis* isolates. The detailed results of the comparative mutational analysis are shown in Table 3.

**Table 3:** The results of the comparative mutational analysis

Drugs	Gene/region	Mutations	No. of isolates (n)
RFP	*ropB*	L511P	1
		D516V	5
		D516Y	2
		D516E	2
		D516G	1
		D516I	1
		N518D	1
		H526Y	4
		H526L	1
		S531L	45
		L533P	2
INH	*katG*	S315T	50
		S315N	2
	*inhA* promoter	-15C → T	6
		-8T → A	1
		-8T → C	1
EMB	*embB*	M306V	25
		M306I	9
		M306L	2

*RFP* rifampicin; *INH* isoniazid; *EMB* ethambutol

Compared with phenotypic DST, the concordance of the SOE PCR and sequencing technique was 94.44%, 94.44%, and 76.85% respectively, for the detection of RFP, INH, and EMB resistance (Table 4). The Kappa value between the SOE PCR and phenotypic DST for EMB resistance was 0.45, indicating moderate agreement, whilst both Kappa values between the SOE PCR and phenotypic DST for RFP and INH resistance detection were 0.89, indicating almost perfect agreement (Table 4). Among the 56 clinical isolates that had GeneXpert MTB/RIF results, the concordance of the SOE PCR and DNA sequencing technique reached 100% for the detection of RFP resistance (Table 5), compared to GeneXpert MTB/RIF. The Kappa value between the SOE PCR and GeneXpert MTB/RIF for RIF resistance was 1, showing almost perfect agreement (Table 5).

**Table 4:** Sensitivity, specificity, PPV, NPV, and agreement of the SOE PCR assay compared with phenotypic DST among *M. tuberculosis* isolates

Drugs	DST	SOE PCR							
		R	S	Sensitivity (%)	Specificity (%)	PPV (%)	NPV (%)	Concordance (%)	Kappa value
RFP	R	58	0	100.00	88.00	90.63	100.00	94.44	0.89
	S	6	44						
INH	R	53	3	94.64	94.23	94.64	94.23	94.44	0.89
	S	3	49						
EMB	R	20	9	68.97	79.75	55.56	87.50	76.85	0.45
	S	16	63						

*S* susceptible; *R* resistant; *PPV* positive predictive value; *NPV* negative predictive value; *DST* drug susceptibility testing; *RFP* rifampicin; *INH* isoniazid; *EMB* ethambutol

**Table 5:** Sensitivity, specificity, PPV, NPV, and agreement of the SOE PCR assay compared with GeneXpert MTB/RIF among *M. tuberculosis* isolates

Drugs	GeneXpert MTB/RIF	SOE PCR							
		R	S	Sensitivity (%)	Specificity (%)	PPV (%)	NPV (%)	Concordance (%)	Kappa value
RFP	R	34	0	100.00	100.00	100.00	100.00	100.00	1.00
	S	0	22						

*S* susceptible; *R* resistant; *PPV* positive predictive value; *NPV* negative predictive value; *DST* drug susceptibility testing; *RFP* rifampicin

## Discussion

In the present study, we constructed an overlap extension PCR diagnostic technique that can rapidly detect drug resistance in *M. tuberculosis*, and we evaluated the application of this method to detect resistance of *M. tuberculosis* to three first-line anti-tuberculous drugs, i.e., RFP, INH, and EMB. Compared with DST, which is considered to be the gold standard for the detecting DR-TB, our SOE PCR method showed high consistence with DST results, with a concordance of 94.44%, 94.44%, 76.85% for resistance to RFP, INH, and EMB respectively. Compared with the GeneXpert MTB/RIF, the SOE PCR method was entirely consistent with GeneXpert MTB/RIF, with a concordance of 100% for resistance to RFP.

The overall turnaround time of our SOE PCR method, including DNA isolation (about 0.5 h), PCR amplification (1.5–2.0 h), sequencing (2.5–3 h), and analysis (10–30 m), is approximately 5–8 h. Although this is not as rapid as the GeneXpert MTB/RIF (about 2 h) [18], the process is comparable with oligonucleotide array (6–7 h) [19], DNA Microarrays (4–5 h) [20] and line probe technology (about 5 h) [20], and is substantially faster than traditional mycobacterial culture and DST, the inherent turnaround time of which is up to several weeks [1].

Resistance to RFP, as the most effective first-line anti-tuberculosis drug, is a key factor in determining the feasibility of a specific TB treatment regimen, and the final treatment outcome. RFP inhibits RNA synthesis, inhibits the reproduction of pathogenic bacteria, and achieves its therapeutic goal through the β subunit of bacterial RNA polymerase (rpoB code) that is dependent on DNA in *M. tuberculosis* [21]. The overwhelming majority of the mutations in the *rpoB* gene occur in an 81 bp RFP-resistance determining region (RRDR), which contributes to over 96% of RFP resistance in *M. tuberculosis* [22]. Therefore, *rpoB* was selected as the target resistant gene for RFP in this study. Moreover, the level of RFP resistance caused by mutations at these different sites of RRDR may be dissimilar, which has important implications for drug dosages prescribed to patients [23]. Compared with other mutations, including the codon 516 and 533 mutation, in our study, mutation of S531L showed a higher prevalence, occurring in 45 of the 64 RFP-resistant specimens. This is highly consistent with other reports in the literature [24–27]. Further, it has been reported that mutation of S531L confers a high level of resistance to RFP (MIC > 32 mg/ml) [23], indicating the significant role of such mutations in RFP resistance and TB treatment. The method employed in the present study directly detects accurate genetic mutational information, which is of great significance for the detection of drug resistance and efficient treatment of TB patients.

INH is another critical first-line medicine used for the treatment of active tuberculosis (TB) as well as latent TB infection [28, 29]. Multiple gene mutations have been associated with INH resistance, most frequently *katG* and the promoter region of the *inhA* gene [30–32]. One systematic review analyzed mutational data from 118 publications in 49 countries, and observed that the most frequently seen mutation was *katG315* (64%), and the second most frequently observed mutation was *inhA* promoter (*inhA-15)* [30]. Other mutations such as *ahpC* promoter gene have also been reported to be one of the indicators for high INH resistance [33]; however, the overall prevalence of the *ahpC* promoter gene mutation is low [34]. Thus, in this study, *katG* and *inhA* promoter were included in the detection indicators of our drug resistance detection method. Our results showed that 50 of the 56 INH-resistant isolates had S315T mutations, and 8 isolates with mutations in the *inhA* promoter sequence were found. Furthermore, increasing reports indicate that RFP-resistant *M. tuberculosis* strains are often resistant to INH as well [35–37], which is consistent with the results of the present study. Our study revealed that 82.8% of RFP resistant isolates (53/64) were simultaneously resistant to INH.

EMB is an arabinose analogue, which can interfere with the synthesis of the bacterial cell wall by inhibition of arabinosyl transferase, compromising the integrity of the cell wall, and thereby causing the death of mycobacteria. Drug resistance to EMB is due mainly to effects related to the biosynthesis of arabinogalactan [38, 39]. This is caused by mutations of genes related to biological activity, and the mutation in position 306 of the gene *embB* is considered to be the main cause of drug resistance [40, 41]. In concordance with this, our results showed that 25 of the 36 EMB resistant specimens had M306V mutations, while 9 were M306I mutations and 2 were M306L mutations. However, the results of the comparison between the SOE PCR method and DST for the detection of EMB resistance were less consistent. It is speculated that EMB resistance may not only be related to *embB* gene mutations. Other unknown molecular mechanisms are speculated to exist. Studies have shown that the efflux pump system also plays an important role in the development of RFP, INH, and EMB resistance [42–44]. Some efflux pump inhibitors have been developed as novel anti-tuberculous drugs in combination with other drugs [45, 46]. Thus, further investigation is warranted to fully reveal the molecular mechanisms underlying specific anti-tuberculous drug resistance, and to construct more efficiently optimized TB drug treatment plans.

The SOE PCR method, which utilizes the sequencing of *rpoB-embB-katG-inhA* fusion fragments in order to detect drug-resistant mutations, has multiple operational clinical advantages. Our method simplifies routine PCR diagnostic methods and sequencing steps, and instead of the need for four separate PCR reactions to amplify each of four separate test tubes containing either *rpoB, embB, katG,* or *inh*, we propose a single PCR reaction to amplify a single test tube of *rpoB-embB-katG-inhA* fusion fragment. Consequently, four sequencing reactions are reduced to one, which greatly reduces workload and increases efficiency of laboratory testing, and also greatly reduces the material cost of testing. Compared with the probe method or the melting curve method, which do not provide specific mutational information, our method can give direct accurate mutational information, and significantly, avoids misreporting of the *rpoB* and *embB* gene synonymous mutations as drug-resistant mutations, as is seen with the probe method and the melting curve method.

We acknowledge that our study has some limitations. Our method, (a) requires a large number of mycobacteria to be present in the sample; (b) cannot be applied directly for clinical sample detection since its sensitivity is not as high as expected when used for the direct detection of DNA in clinical sputum specimens (*M. tuberculosis* DNA needs to be extracted from clinical specimens prior to detection); (c) is more time-consuming compared with the GeneXpert assay; (d) requires manual analysis of test results.

## Conclusions

We have successfully developed a novel SOE PCR diagnostic method, and this method is potentially useful for rapidly detecting resistance to RFP, EMB, and INH simultaneously in *M. tuberculosis* isolates. Our method has undergone a validation process, and was found to show high specificity, sensitivity, and concordance when compared to DST or GeneXpert MTB/RIF. Our novel TB drug resistance detection method may be used as an auxiliary diagnostic drug resistance testing method, and effectively circumvents the sometimes onerous limitations of current detection technologies in clinic, especially in regions which lack the availability of high-cost methods and specialized or dedicated laboratory facilities and personnel, such as the GeneXpert MTB/RIF assay, the DNA microarray system, and gene chip technology.

## Supplementary Information

**Additional file 1.** The DNA sequence of the* rpoB-embB-katG-inhA* fusion fragment.

**Additional file 2.** The amino acid sequence associated with the* rpo-BembB-katG-inhA* fusion fragment.

## Data Availability

The datasets used and/or analyzed during the present study are available from the corresponding author upon reasonable request.

## Abbreviations

DR-TB: Drug-resistant tuberculosis
DST: Drug susceptibility testing
EMB: Ethambutol
INH: Isoniazid
MDR-TB: Multidrug-resistant tuberculosis
RFP: Rifampicin
RR-TB: Rifampicin-resistant tuberculosis
SOE PCR: Gene splicing by overlap extension PCR
TB: Tuberculosis
