# Supplementary material for: Establishment and evaluation of an overlap extension polymerase chain reaction technique for rapid and efficient detection of drug-resistance in Mycobacterium tuberculosis
Source: Infect Dis Poverty. 2022 Mar 24;11:31. doi: 10.1186/s40249-022-00953-5 (PMC8942611; doi:10.1186/s40249-022-00953-5)
Supplement: Supplementary file 1 — Additional file 1. The DNA sequence of the rpoB-embB-katG-inhA fusion fragment. [file 40249_2022_953_MOESM1_ESM.doc]

The DNA sequence of the rpoB (1–273 bp)-embB (274–420 bp)-katG (421–564 bp)-inhA (565–722 bp) fusion fragment obtained by the SOE PCR technique was the following:

1 CAAAACCAGA TCCGGGTCGG CATGTCGCGG ATGGAGCGGG TGGTCCGGGA

51 GCGGATGACC ACCCAGGACG TGGAGGCGAT CACACCGCAG ACGTTGATCA

101 ACATCCGGCC GGTGGTCGCC GCGATCAAGG AGTTCTTCGG CACCAGCCAG

151 CTGAGCCAAT TCATGGACCA GAACAACCCG CTGTCGGGGT TGACCCACAA

201 GCGCCGACTG TCGGCGCTGG GGCCCGGCGG TCTGTCACGT GAGCGTGCCG

251 GGCTGGAGGT CCGCGACGTG CACGCGGTGA TATTCGGCTT CCTGCTCTGG

301 CATGTCATCG GCGCGAATTC GTCGGACGAC GGCTACATCC TGGGCATGGC

351 CCGAGTCGCC GACCACGCCG GCTACATGTC CAACTATTTC CGCTGGTTCG

401 GCAGCCCGGA GGATCCCTTC GCGTATGGCA CCGGAACCGG TAAGGACGCG

451 ATCACCAGCG GCATCGAGGT CGTATGGACG AACACCCCGA CGAAATGGGA

501 CAACAGTTTC CTCGAGATCC TGTACGGCTA CGAGTGGGAG CTGACGAAGA

551 GCCCTGCTGG CGCTAGCGTA ACCCCAGTGC GAAAGTTCCC GCCGGAAATC

601 GCAGCCACGT TACGCTCGTG GACATACCGA TTTCGGCCCG GCCGCGGCGA

651 GATGATAGGT TGTCGGGGTG ACTGCCACAG CCACTGAAGG GGCCAAACCC

701 CCATTCGTAT CCCGTTCAGT CC
