# Supplementary material for: Establishment and evaluation of an overlap extension polymerase chain reaction technique for rapid and efficient detection of drug-resistance in Mycobacterium tuberculosis
Source: Infect Dis Poverty. 2022 Mar 24;11:31. doi: 10.1186/s40249-022-00953-5 (PMC8942611; doi:10.1186/s40249-022-00953-5)
Supplement: Supplementary file 2 — Additional file 2. The amino acid sequence associated with the rpo-BembB-katG-inhA fusion fragment. [file 40249_2022_953_MOESM2_ESM.doc]

The amino acid sequence associated with the rpoB-embB-katG-inhA fusion DNA fragment sequence of the standard strain H37Rv was as follows (inhA promoter encodes no amino acid):

1 QNQIRVGMSR MERVVRERMT TQDVEAITPQ TLINIRPVVA AIKEFFGTSQ

51 LSQFMDQNNP LSGLTHKRRL SALGPGGLSR ERAGLEVRDV HAVIFGFLLW

101 HVIGANSSDD GYILGMARVA DHAGYMSNYF RWFGSPEDPF AYGTGTGKDA

151 ITSGIEVVWT NTPTKWDNSF LEILYGYEWE LTKSPAGA
